# Polyphyletic domestication and inter-lineage hybridization magnified genetic diversity of cultivated melon, *Cucumis melo* L.

**DOI:** 10.1270/jsbbs.24045

**Published:** 2025-06-10

**Authors:** Katsunori Tanaka, Gentaro Shigita, Tran Phuong Dung, Phan Thi Phuong Nhi, Mami Takahashi, Yuki Monden, Hidetaka Nishida, Ryuji Ishikawa, Kenji Kato

**Affiliations:** 1 Faculty of Agriculture and Life Science, Hirosaki University, 1 Bunkyo, Hirosaki, Aomori 036-8561, Japan; 2 Graduate School of Environmental and Life Science, Okayama University, 1-1-1 Tsushima-Naka, Kita-ku, Okayama 700-8530, Japan; 3 University of Agriculture and Forestry, Hue University, No. 102 Phung Hung Street, Hue City, 49000 Vietnam; 4 Graduate School of Environmental, Life, Natural Science and Technology, Okayama University, 1-1-1 Tsushima-Naka, Kita-ku, Okayama 700-8530, Japan; 5 Department of Life Science Systems, Technical University of Munich, Emil-Ramann Strasse 2, Freising 85354, Germany

**Keywords:** chloroplast genome, *Cucumis melo*, domestication, genetic diversity, melon, molecular polymorphism, seed size

## Abstract

Melon accessions with diverse geographical origins were classified into large and small seed-types by length of seed at the boundary of 9 mm, and into five populations based on polymorphisms in the nuclear genome. They were further divided into three maternal lineages, Ia, Ib, and Ic, by polymorphisms in the chloroplast genome. By combining these three classifications, the Europe/US subsp. *melo* and the East Asian subsp. *agrestis* were characterized as [large seed, Ib, PopA1 or A2] and [small seed, Ia, PopB1 or B2], respectively, indicating nearly perfect divergence. In South Asia, in addition to the Europe/US and East Asian types, recombinant types between the two types were detected and accounted for 34.8% of South Asian melon. The finding of such an intermixed structure of genetic variation supported the Indian origin of Ia and Ib types. As to Momordica popular in South Asia, seed length was intermediate between the large and small seed-types, and chloroplast type was a mixture of Ia and Ib, suggesting its origin from the recombinant type. In Africa, three lineages of melon were distributed allopatrically and showed distinct divergence. Subsp. *agrestis* of the Ic type proved to be endemic to Africa, indicating its African origin.

## Introduction

Melon (*Cucumis melo* L.) is cultivated worldwide. Its history of cultivation can be dated back to the 3rd–2nd millennia BCE in Africa and the Asian continent (Kirkbride 1993, Walters 1989, Zohary and Hopf 2000). Reflecting this long history of cultivation, diverse types of cultivated melon have been established in various parts of the world, and melon is considered the most diversified species of Cucurbitaceae. It is separated into two subspecies, *melo* L. and *agrestis* Naudin (Naudin 1859), the former being typically grown in Europe and the US and the latter in East and South Asia. As summarized by Pitrat (2016, *C. melo* is divided into 19 horticultural groups based primarily on flower and fruit traits: Agrestis, Kachri, Chito, Tibish, Acidulus, Momordica, Conomon, Makuwa, Chinensis, Flexuosus, Chate, Dudaim, Chandalak, Indicus, Ameri, Cassaba, Ibericus, Inodorus, and Cantalupensis.

Previous studies of genetic diversity among melon accessions from diverse geographical origins highlighted three points concerning the evolution and diversification of melon, though the direct ancestral wild species has yet to be identified. The first point is the genetic divergence between Europe/US melons, Cantalupensis and Inodorus, and East Asian melons, Conomon and Makuwa, as demonstrated by analysis of seed size (Fujishita 1983, Sabato *et al.* 2015, Tanaka *et al.* 2016), fruit characteristics (Liu *et al.* 2004), molecular markers (Esteras *et al.* 2013), genome-wide Single Nucleotide Polymorphisms (SNPs) (Liu *et al.* 2020, Shigita *et al.* 2023, Wang *et al.* 2021, Zhao *et al.* 2019) and both phenotypic traits and molecular markers (Stepansky *et al.* 1999, Tanaka *et al.* 2013). The Europe/US melon is characterized by sweetness of the fruit flesh and seeds longer than 9.0 mm, the East Asian melon by less sweetness of the fruit flesh and seeds shorter than 9.0 mm. Sequence polymorphisms of the chloroplast genome, including SNPs, In/Del, and Simple Sequence Repeats (SSRs), were detected by Tanaka *et al.* (2013) and suggested that the Europe/US melon subsp. *melo* and the East Asian melon subsp. *agrestis* were established in different maternal lineages, Ib and Ia, respectively. This finding strongly supported the independent origin of cultivated melon (Endl *et al.* 2018, Pitrat 2013, Zhao *et al.* 2019). However, most of the studies focused on either of morphological traits (e.g., seed size), nuclear genome or chloroplast genome, and thus comprehensive study is required to understand genetic differentiation among the two geographical groups of melon.

The second point is genetic structure of Indian melon. India had been considered the secondary center of diversity of cultivated melon (Robinson and Decker-Walters 1997), and higher phenotypic and genetic variations have been reported (Akashi *et al.* 2002, Dhillon *et al.* 2007, 2012, Haldhar *et al.* 2018, McCreight *et al.* 2004, Tanaka *et al.* 2007). Tanaka *et al.* (2013) analyzed the chloroplast genome and seed length of 60 melon accessions with diverse geographical origins, including nine accessions of Indian landraces, and indicated the presence of the Europe/US type (large seed-type with Ib cytoplasm) and the East Asian type (small seed-type with Ia cytoplasm) in India. In addition, recombinant types between two types, e.g., large seed-type with Ia cytoplasm and small seed-type with Ib cytoplasm, were also found. Recombinants could be established by hybridization between the two types, and part of the recombinants might differentiate into Flexuosus and Momordica. However the number of accessions studied was limited, and thus further detailed studies using more landraces including Momordica and Flexuosus is required, to figure out the evolution and diversification of melon in India.

The third point concerns the African origin of melon. Africa is thought to be another center of melon domestication (Kirkbride 1993, Pitrat 2008, Zohary and Hopf 2000). Analyses using Random Amplified Polymorphic DNA (RAPD) and/or SSR markers revealed large genetic variation in African melon (López-Sesé *et al.* 2002, Mliki *et al.* 2001, Serres-Giardi and Dogimont 2012). Landraces from Northern Africa are closely related to those in Europe/US (Mliki *et al.* 2001, Nakata *et al.* 2005), while those from South and Western Africa are closely related to those in India and East Asia (Esteras *et al.* 2013, Monforte *et al.* 2003). Diversity in African melon was also demonstrated by analysis of the chloroplast genome, and all three cytoplasm types, Ia, Ib and Ic, were detected in 29 landraces examined (Tanaka *et al.* 2013). The Ia and Ib types accord with those in East Asia and in Europe/US, respectively. In contrast, the Ic type was found only in Western and Southern Africa and was rather distantly related to the other two types, suggesting the independent origin of the Ic type in the third lineage in Africa. To validate spatial genetic differentiation in Africa and its correspondence with that on other continents, the genetic structure of a worldwide collection of melon should be examined.

Here, we revealed the genetic structure of Indian melon which consists of two groups of melon, subsp. *melo* and subsp. *agrestis*, and recombinant types between the two groups, based on analyses of seed size, nuclear genotype and cytoplasm type, using 212 melon accessions with diverse geographical origins including 73 accessions from South Asia. We also discussed the genetic differentiation between three maternal lineages of melon.

## Materials and Methods

### Plant materials

A total of 212 accessions of melon (*Cucumis* *melo* L.), selected from a wide geographical area of Africa, Asia, Europe and North America were analyzed. They consisted of 31 accessions of Agrestis, 84 of six horticultural groups classified after Pitrat (2016), and 97 unclassified landraces. The geographical origin and horticultural group of each accession are given in Supplemental Table 1. Since distinct seed size variation within accession was observed in two accessions from India, PI 210541 and PI 124112, they were separated into two accessions. Two accessions of Japanese Cantalupensis and one accession of Chinese Honeydew were included in the Europe/US group for analysis, since they derived from recent introductions from Europe or the USA. Five accessions of the Chinese Hami melon were included in the West and Central Asian group, since it is distantly related to the Chinese Conomon and Makuwa and closely related to West and Central Asian melon (Aierken *et al.* 2011).

Seeds of these accessions were provided by the North Central Regional Plant Introduction Station, Iowa State University (USDA-ARS), USA; the NARO Institute of Vegetable and Floriculture Science (NIVFS), Japan; and Okayama University, Japan. These accessions were cultivated in the field or greenhouse of Okayama University, and self-pollinated to harvest mature seeds for size measurement.

### Seed size measurement

The lengths and widths of three representative seeds were measured for each accession. Based on seed length, each accession was classified into the large seed-type (≥9.0 mm) or small seed-type (<9.0 mm) according to Akashi *et al.* (2002).

### DNA extraction

For each accession, genomic DNA was extracted from a single ten-day-old seedling, after Murray and Thompson (1980), with minor modifications.

### Chloroplast genotyping

Chloroplast genome type was determined by analysis of nine SNP markers, one In/Del marker and two SSR markers (Table 1). Of these 12 markers, ccSSR7 was developed by Chung and Staub (2003) and the others were newly developed in this study from sequence polymorphisms previously detected by Tanaka *et al.* (2013).

Nine SNPs were examined by three Cleaved Amplified Polymorphic Sequence (CAPS) and six derived CAPS (dCAPS) markers (Table 1). PCR amplification was done by using *Taq* DNA polymerase (Sigma-Aldrich^®^, USA), and the PCR product was digested with the restriction enzymes shown in Table 1. The digested product was electrophoresed in agarose gel for SNP genotyping. For PCR amplification of PSID-dCAPS1 and PSID-dCAPS2, PSID-R primer was used as a common reverse primer. For genotyping ccSSR-7, the deletion of 5 bp was detected by acrylamide gel electrophoresis according to Aierken *et al.* (2011).

For Cmcp3-SSR and PSID-SSR, PCR amplification was done using Ex*Taq*^TM^ DNA polymerase (Takara, Japan). The PCR product, including a fluorescently labeled size marker, was applied to a CEQ8000 capillary sequencer (Beckman Coulter, USA). The resulting chromatograms were visualized and analyzed using CEQ 8000 Fragment Analysis software.

Additional sequence polymorphisms were indicated by the analysis of Cmcp11-dCAPS, Cmcp3-SSR and PSID-SSR. The Cmcp11 region was sequenced for five accessions (PI 185111, 940101, 940103, 770134, and 770135), and Cmcp3 and PSID regions for three accessions (940099, 940102, and PI 614519). The experimental procedure is fully described by Tanaka *et al.* (2013), and the representative nucleotide sequences determined were registered in the DNA Data Bank of Japan (DDBJ) (Supplemental Table 2).

### RAPD analysis

RAPD analysis was carried out for 147 melon accessions. Eighteen random primers (12-mer, Bex, Japan) selected for their ability to detect polymorphism by Tanaka *et al.* (2007) were used to produce 27 markers (Supplemental Table 3). PCR amplification and electrophoresis were carried out according to Tanaka *et al.* (2007). For the remaining 65 accessions, RAPD data obtained by Tanaka *et al.* (2007) was used for data analysis.

### Data analysis

Tanaka *et al.* (2013) identified a total of 12 subtypes of the chloroplast genome, and three additional subtypes, Ia-4, Ia-5, and Ia-6, were detected in this study. To investigate the relationship among 15 cytoplasm types, a Median-Joining (MJ) network (Bandelt *et al.* 1999) was constructed with the NETWORK program, v4.6.1.1. For the calculation, we used sequence data of 30 SNPs (SNP 1–30) and five In/Del (InDel 1–5) of three accessions sequenced in this study (940099, 940102, and PI 614519) and of 53 accessions including four wild species, *C. anguria*, *C. hystrix*, *C. metuliferus* and *C. sagittatus*, previously sequenced by Tanaka *et al.* (2013). The superfluous median vector on the constructed network tree was purged by the maximum parsimony option (Polzin and Daneschmand 2003). The same data set was used to construct neighbor-joining (NJ) and maximum-likelihood (ML) trees with a bootstrapping of 1,000 replications using MEGA v11 (Tamura *et al.* 2021).

Marker bands of RAPD were scored as 1 for a positive band and zero for a null band. The number of different alleles (Na), number of effective alleles (Ne), and expected heterozygosity (He) were calculated using GenALEX v6.503 (Peakall and Smouse 2006, 2012). The polymorphic information content (PIC) and gene diversity (*D*) within each group were then calculated according to Botstein *et al.* (1980) and Nei (1972). The genetic distance (GD) among accessions was calculated as described by Apostol *et al.* (1993). Based on the GD matrix, a dendrogram was constructed by using the Unweighted Pair Group Method with arithmetic mean (UPGMA), using PHYLIP v3.698 programs (https://evolution.genetics.washington.edu/phylip.html), and was compared with that by the NJ method. The relationships among melon groups were visualized by principal coordinate analysis (PCO) and quantified by fixation index (*F_ST_*) value by GenALEX v6.503. The model-based clustering program, STRUCTURE v2.3.4 (Pritchard *et al.* 2000), was used to infer population structure by a Bayesian approach from the RAPD marker data set. The optimal value of *K* (the number of clusters) was deduced by evaluating *K* = 1–10, and determined by an admixture model with an allele frequencies correlated model. The length of burn-in of the Markov Chain Monte Carlo (MCMC) iterations was set to 5,000 and data were collected over 5,000 MCMC iterations in each run. Twenty iterations per *K* were conducted. The optimal value of *K* was identified using the ad hoc procedure introduced by Pritchard *et al.* (2000) and the method developed by Evanno *et al.* (2005), which were carried out in Structure Harvester (Earl and vonHoldt 2012). Data plotting after the STRUCTURE simulation was conducted with CLUMPP (Jakobsson and Rosenberg 2007). Substructures within each major population were detected by repeating the simulation for each population with the same settings.

## Results

### Seed size measurement

**Fig. 1.**
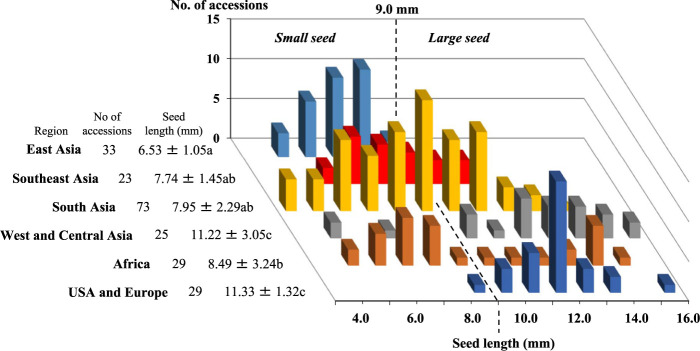
Frequency distribution of seed length in melon accessions from six geographical regions. Geographical differences were analyzed by the Tukey-Kramer multiple comparison test with significant differences at p < 0.01.

Average seed length showed wide intraspecific variation, ranging from 3.9 mm to 15.1 mm among the 212 accessions, as shown in Fig. 1, and average seed widths ranged from 1.8 mm to 7.0 mm. These two traits were closely correlated (*r* = 0.912, *p* < 0.01), and thus seed length was used as an indicator of seed size hereafter. The difference in seed length between geographical regions was significant (*p* < 0.01), though it was insignificant between Europe/US and West and Central Asia, and between South Asia and Southeast Asia. The analysis of seed length classified the Europe/US accessions into the large seed-type (≥9.0 mm) except for one Japanese netted accession, Melon Chuukanbohon Nou 1, bred by crossing Cantalupensis and Makuwa, while the East Asian accessions were classified as small seed-type (<9.0 mm). Among melon accessions from other regions, those from Northern Africa and West Asia were recognized as large seed-type. In contrast, the small seed-type predominated in Western and Southern Africa. These results demonstrate geographical differentiation in seed length.

Seed lengths showed continuous variation in South and Southeast Asia, where both large and small seed-types were frequently found. In these regions, two classes (8–9 mm and 9–10 mm), flanking the boundary between the large and small seed-types, accounted for 30.2% of accessions, including 11 of the 22 accessions of Momordica and Flexuosus, whose average seed lengths were 9.3 mm and 9.7 mm, respectively. In contrast, the frequency of the two classes was low, being 6.1% in East Asia and 13.8% in Europe/US.

### Chloroplast genome type

**Fig. 2.**
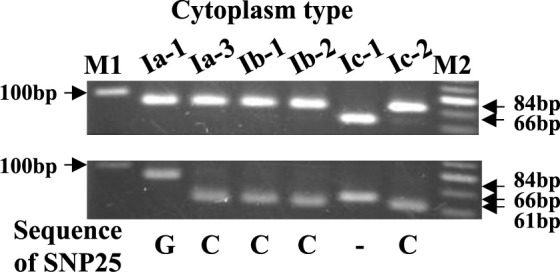
Gel image of dCAPS analysis of Cmcp11-dCAPS. Agarose gel electrophoresis patterns of amplified products (upper panel) and restriction fragments digested with *Rsa* I (lower panel). M1; 100 bp DNA Ladder (Takara, Japan), Ia-1; ‘Kinpyo’, Ia-3; PI 482424, Ib-1; 525105, Ib-2; ‘Earl’s Favourite’, Ic-1; PI 185111, Ic-2; PI 436533, M2; 20 bp DNA Ladder (Takara, Japan).

The sequence polymorphisms identified by Tanaka *et al.* (2013) were detected by PCR analysis of CAPS, dCAPS, and SSR markers of the chloroplast genome (Fig. 2). As summarized in Table 2, new chloroplast genome subtypes Ia-4 to Ia-6 were detected in Agrestis from South Asia, with unique combinations of Cmcp11-dCAPS, Cmcp3-SSR and PSID-SSR alleles (Supplemental Table 1). In contrast, Ic-3 and Ic-4 could not be distinguished, since the diagnostic marker for SNP21 was not used in this study. Likewise, Ic-5 and Ic-6 were not distinguished, since the diagnostic marker for InDel2 was not used. Of the 212 accessions, 118, 82, and 12 were classified as Ia, Ib, and Ic types, respectively, and were further divided into 13 subtypes (Table 2).

**Fig. 3.**
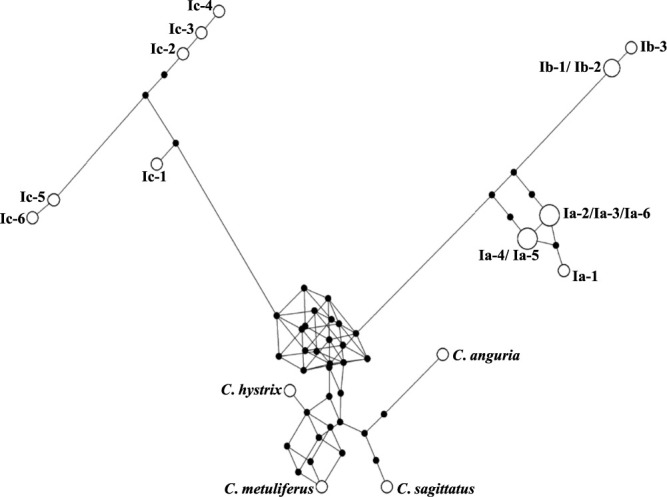
MJ networks (ε = 10) of 19 types of chloroplast genome in *Cucumis*. White open circles indicate each chloroplast type. The sequence polymorphisms on the link (SNP1-35, InDel 1–5) refer to mutated nucleotides. Sequence types indicated by black solid circles are added for growing network as median vectors.

A phylogenetic relationship of the three cytoplasm types, Ia, Ib and Ic, was demonstrated by the MJ network in which four wild species of *Cucumis* were included as outgroups (Fig. 3). A total of 15 subtypes detected in *C. melo* formed three distinct groups with no reticulation among them (i.e., no intermediates between the three groups). Therefore, the three cytoplasm types were distinctly differentiated from each other. In the MJ network, the Ia and Ib types were allocated in the same lineage, with the Ia type positioned closer to the outgroups, suggesting that it was cultivated earlier than the Ib type and that the Ib type evolved later. On the other hand, the Ic type evolved from wild species of *Cucumis* in another lineage. Interestingly, two subtypes, Ic-5 and Ic-6, were found in Ghana and Senegal, in the west of the prime meridian, while the other four subtypes of Ic were in the east. The tree topology, “(Ic, (Ib,Ia))”, was also confirmed in the NJ and ML trees (Supplemental Fig. 1).

### Nuclear marker genotyping

Eighteen RAPD primers amplified 27 polymorphic marker bands of approximately 550–2027 bp, with an average of 1.7 effective marker bands produced by each primer (Supplemental Table 3). PIC varied depending on the primer, and ranged from 0.044 to 0.282.

Gene diversity (*D*) ranged from 0.147 to 0.367 in 10 melon groups classified based on geographical origin and seed length type (Supplemental Table 4). The largest diversity (*D* = 0.367) was observed in the large seed-type of South Asia. For small seed-type melon, the largest diversity (*D* = 0.292) was observed also in South Asia. In both large and small seed-types, the *D* values gradually decreased from South Asia towards Central and West Asia and Europe on the west, and towards Southeast Asia and East Asia on the east (Table 3).

**Fig. 4.**
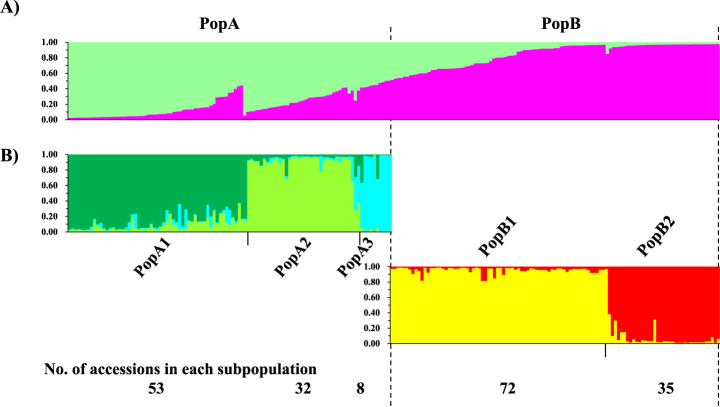
Classification of 212 melon accessions by STRUCTURE simulation based on RAPD and SSR polymorphisms. A) Optimal population structure (*K* = 2). B) Subpopulation structure of PopA (*K* = 3) and PopB (*K* = 2). Each single vertical line represents a melon accession, and different colors represent different populations and subpopulations. The length of the colored segment illustrates the estimated proportion of each accession’s membership in the corresponding groups.

LnP(D) values from the STRUCTURE analysis increased with *K* from 2 to 10, with an evident inflection at *K* = 2 (Supplemental Fig. 2A). According to second-order statistics, to estimate the number of subpopulations, the optimal value of *K* = 2 was identified, at which Delta *K* (Evanno *et al.* 2005) showed a peak. These results indicate that the diverse melon accessions used in this study consisted of two populations, designated PopA and PopB (Fig. 4A). Accessions with an estimated membership of over 0.5 were assigned to the respective population. Subpopulation structuring under PopA and PopB was performed by repeating the simulation for each population, and 12 accessions with estimated memberships below 0.7 were assigned to the “admixed group” (Supplemental Table 1). PopA was divided into three subgroups (*K* = 3) and PopB into two subgroups (*K* = 2) (Fig. 4B, Supplemental Fig. 2B, 2C). Consequently, the 212 accessions were divided into five subgroups designated as PopA1, PopA2, PopA3, PopB1, and PopB2, except for the 12 accessions of the admixed group. Genetic divergence, estimated by genetic distance and *F_st_* value, was rather small between PopA1 and PopA2 as well as PopB1 and PoB2, while PopA3 was highly divergent from the other populations (Supplemental Table 5).

**Fig. 5.**
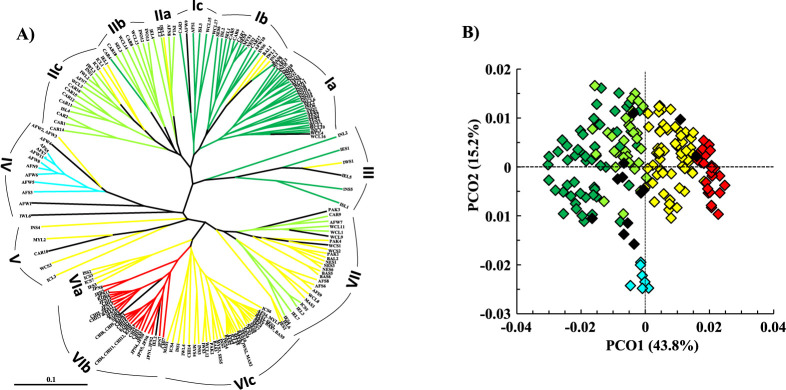
Genetic relationships of 212 melon accessions inferred from RAPD and SSR polymorphisms. A) UPGMA tree of 212 melon accessions. Cluster ID is indicated by Roman numerals. Colors indicate the STRUCTURE-based subpopulations: Green = PopA1, light green = Pop A2, light blue = Pop A3, yellow = PopB1, red = PopB2, black = admixture. B) Distribution of the STRUCTURE-based subpopulations on the first two principal co-ordinates. Colors indicate subpopulations as mentioned above.

An unrooted UPGMA tree of 212 accessions illustrated genetic relationships that closely approximated the STRUCTURE-based membership assignment for most accessions (Fig. 5A). For three subgroups, PopA1, PopA3, and PopB2, accessions of each subgroup formed the respective cluster(s), while accessions of PopA2 and PopB1 were classified into several clusters together with those of other subgroups. Close relationships were again demonstrated between PopA1 and PopA2, and between PopB1 and PopB2. A PCO was conducted to assess the adequacy of population subdivisions by STRUCTURE (Fig. 5B). Plotting of the first two principal components showed separation of inferred subpopulations, which was highly consistent with STRUCTURE-based membership assignment, and showed relationships of these subpopulations, which was consistent with the UPGMA tree. The relationships of all five subpopulations suggested by the UPGMA tree and PCO were also concordant with the GD and *F_ST_* (Supplemental Table 5).

### Classification based on seed length, chloroplast genome and nuclear genome

A total of 212 accessions were consequently classified into several groups as follows: two groups by seed length (Fig. 1), three groups by chloroplast DNA polymorphism (Fig. 3), and five populations by nuclear DNA polymorphism (Fig. 4). If these three classifications are genetically independent, then at most 30 types are possible by combining three classifications. However, 200 of the 212 accessions (excluding admixed groups) were assigned to only 17 types, of which three types consisted of only one accession, suggesting non-random association between three classifications (Table 3). For simplicity, the results of the 200 accessions are indicated hereafter, unless otherwise stated.

Among 61 accessions from Europe/US and East Asia, 59 (96.7%) were classified into four contrasting types: [large seed, Ib, PopA1 or A2] and [small seed, Ia, PopB1 or B2], highlighted in Table 3. Intermediate or recombinant types which could be derived from hybridization between those four types were less frequent. These results show distinct genetic differentiation between the Europe/US large seed-type and the East Asian small seed-type. Although many Europe/US and West and Central Asian melons were classified into two major types [large seed, Ib, PopA1 or A2], the subtype classified differed among horticultural groups, with European Cantalupensis as [Ib-1, PopA1 or A2] and British Cantalupensis and Chinese Hami melon of Inodorus as [Ib-2, PopA1] (Supplemental Table 1). Within the USA, Honeydew melon of Inodorus was characterized by [Ib-3, PopA1] and Cantalupensis mostly by [Ib-2 or Ib-3, PopA2].

Four contrasting types, [large seed, Ib, PopA1 or A2] and [small seed, Ia, PopB1 or B2], were also commonly found in South Asia (Table 3, Supplemental Table 1). However, recombinant types between these four contrasting types were rather frequent (34.8%), and accessions of large and small seed-types consisted of the respective six types. Among the 24 recombinants, 5 (20.8%) were classified as [large seed, Ia, PopA1 or A2], having the genetic characteristics of both the large seed-type (PopA1 or A2) and the small seed-type (Ia). In addition, the frequencies of recombinant types were 14 among 23 accessions (60.9%) in the large seed-type, and 10 among 46 accessions (21.7%) in the small seed-type. These results indicated that recombinant types were found more frequently in the large seed-type than in the small seed-type. Interestingly, of the 14 recombinant accessions of the large seed-type, 12 were classified as either Momordica (7 accessions) or Flexuosus (5).

The unique Ic cytoplasm, which was not found outside Africa, was found frequently in Africa (41.4%) (Table 3). The unique type [small seed, Ic, PopA3] accounted for 24.1% of African accessions and 38.9% of African small seed-type. In addition, three accessions of admixed groups proved to be [small seed, Ic, admixture PopA1/A3] and commonly shared the membership of PopA3. Three contrasting types commonly found worldwide, [large seed, Ib, PopA1 or A2] and [small seed, Ia, PopB1], were also detected (10 and five accessions, respectively). These results indicated that four types of melon, including the unique Ic type, are cultivated or grow in Africa, and that they are differentiated in chloroplast genome in concordance with the nuclear genome. More interestingly, these four types were found allopatrically: eight of 10 accessions of [large seed, Ib, PopA1 or A2] were in Northern Africa, all accessions of [small seed, Ia, PopB1] were in Southern Africa, and five of seven accessions of [small seed, Ic, PopA3] were in Western Africa.

## Discussion

Genetic relationships and divergence among geographical populations and horticultural varieties have been studied in melon, and it is widely accepted that the Europe/US melon (subsp. *melo*) and East Asian melon (subsp. *agrestis*) are genetically divergent in the nuclear genome (Blanca *et al.* 2011, Deleu *et al.* 2009, Esteras *et al.* 2013, Liu *et al.* 2020, Monforte *et al.* 2003, Sabato *et al.* 2015, Shigita *et al.* 2023, Silberstein *et al.* 1999, Wang *et al.* 2021, Zhao *et al.* 2019), and also in the chloroplast genome (Endl *et al.* 2018, Tanaka *et al.* 2013, Zhao *et al.* 2019, Zhu *et al.* 2016). In the present study we analyzed sequence polymorphism of the chloroplast genome by a PCR-based method, and identified three cytoplasm types, Ia, Ib, and Ic (Table 2), as previously reported (Tanaka *et al.* 2013). Zhao *et al.* (2019) analyzed genomic variation by whole genome resequencing and classified 1,175 melon accessions into three clades, *agrestis* clade, *melo* clade, and African clade, based on chloroplast SNPs. These three clades corresponded well to the three cytoplasm types reported by Tanaka *et al.* (2013). Zhu *et al.* (2016) classified seven melon accessions into two chloroplast genome groups by the analysis of 63 SNPs, of which six were identical to the SNP markers analyzed in this study (SNPs 2, 8, 18, 19, 25 and 29) (Table 2). Since the Ic type accession of Africa was not included in Zhu *et al.* (2016), seven accessions were separated into two clades, likely corresponding to Ia and Ib. Therefore, it was confirmed that the 12 markers (Table 1) developed in this study are applicable to the classification of chloroplast genome type, and that the set of markers, Cmcp1-dCAPS, Cmcp6-dCAPS, and Cmcp11-CAPS2, is sufficient to classify the three major cytoplasm types, Ia, Ib, and Ic.

For the Europe/US melon and East Asian melon, all accessions of Europe/US were classified as [large seed, Ib, PopA1 or A2] except for one Japanese breeding line, while 32 of 33 accessions of East Asia were classified as [small seed, Ia, PopB1 or B2] (Table 3). In addition, recombinant types between the four contrasting types were rarely found in these areas, indicating nearly perfect divergence in both nuclear and chloroplast genomes between two peripheral areas of melon distribution. These results support independent origin of subsp. *melo* of Europe/US and subsp. *agrestis* of East Asia (Zhao *et al.* 2019). A large genetic diversity of melon proved to be partly ascribable to its polyphyletic origin.

In contrast, less attention has been paid to cultivated melon in South Asia and only little is known about their maternal lineage, despite India being known as the secondary center of diversity of cultivated melon (Akashi *et al.* 2002, Robinson and Decker-Walters 1997, Tanaka *et al.* 2007) and also claimed as the place of melon domestication (Endl *et al.* 2018, John *et al.* 2013, Sebastian *et al.* 2010, Zhao *et al.* 2019). In South Asia, both large and small seed-types are commonly found, and seeds length varied continuously from 3.9 mm to 13.2 mm (Fig. 1). More importantly, accessions with seed lengths from 8 mm to 10 mm, flanking the boundary between large and small seed-types, accounted for 31.5%, which was higher than those in Europe/US (14.7%) and East Asia (6.0%). The abundance of accessions having intermediate-sized seeds was also indicated by the analysis of 1,998 Indian accessions of USDA-ARS GRIN (Tanaka *et al.* 2013). In this context, Momordica and Flexuosus included 15 accessions of large seed-type and 7 accessions of small seed-type (Supplemental Table 1), and their seed lengths showed continuous variation from 5.7 mm to 13.2 mm.

The proportions of four types, [large seed, Ib, PopA1 or A2] of subsp. *melo* and [small seed, Ia, PopB1 or B2] of subsp. *agrestis* were low in South Asia compared with those in Europe/US and East Asia, being 39.1% in the large seed-type and 77.8% in the small seed-type, showing the abundance of recombinant/intermediate types. These results indicated that South Asia is rich in genetic diversity of seed length and nuclear and chloroplast genomes (Fig. 1, Supplemental Table 4), as reported previously (Akashi *et al.* 2002, Dhillon *et al.* 2007, Gonzalo *et al.* 2019, Shigita *et al.* 2023, Tanaka *et al.* 2007). Genetic variation is expected to be large simply by the coexistence of different types of melon, including Momordica, even if they are reproductively isolated from each other. However, genetic interchange through spontaneous hybridization between subsp. *melo* and subsp. *agrestis* seems to be common in India, especially in the large seed-type. As a result, polymorphisms of seed length and nuclear and chloroplast genomes are not closely associated, unlike the Europe/US subsp. *melo* and East Asian subsp. *agrestis* (Table 3). The presence of various kinds of recombinant/intermediate types made their botanical classification difficult, and supported the practical classification into horticultural groups proposed by Pitrat (2016). The Indian origin of melon with Ia and Ib cytoplasms was also affirmed by such an intermixed structure of genetic variation. Although several species of *Cucumis* was reported as wild in India (Naudin 1859), most of them have been regarded as feral type (Agrestis group) and reclassified as *C. melo* (Kirkbride 1993). However, collection and detailed analysis of living samples, as well as perusal of herbarium specimens, revealed the presence of wild species of *Cucumis* with somatic chromosome number 2n = 24, i.e., *C. callosus* (John *et al.* 2013), *C. setosus* (John *et al.* 2014), and *C. silentvalleyi* (John *et al.* 2017). Of these, *C. callosus* can produce fertile hybrid with cultivated melon, and is considered as the candidate of the ancestoral species of melon with Ia and Ib cytoplasms.

Momordica is mainly grown in India and Southeast Asia, and is characterized by unique characteristics such as mealy flesh, very thin exocarp splitting at maturity, and so on (Dhillon *et al.* 2007, Pitrat 2016). Interestingly, Momordica (locally called Shimauri or Babagoroshi) is cultivated on Hachijojima Island in Japan, a remote island located 287 km south of Tokyo, and shows the same characteristics as Indian Momordica. Based on the length of ancient melon seed remains, Fujishita (2008) and Tanaka *et al.* (2015) indicated that Momordica was commonly grown in Japan from the 4th to the 11th century. These results suggest wide distribution of Momordica in Asia in the past, though it is now limited to South and Southeast Asia. Of eight accessions of Indian Momordica examined in the present study, four (50.0%) proved to be the recombinant type. Both Ia and Ib types were also detected. These results, together with seed length variation, suggest that Momordica might have originated from the reciprocal hybrids between [large seed, Ib, PopA1/A2] and [small seed, Ia, PopB1/B2] types.

Africa is considered to be one domestication center of melon (Endl *et al.* 2018, Kirkbride 1993, Pitrat 2008, Zhao *et al.* 2019, Zohary and Hopf 2000). As shown in Table 3 and Supplemental Table 1, allopatric distribution of three groups of melon was confirmed in Africa; [large seed, Ib, PopA1 or A2], [small seed, Ia, PopB1], and [small seed, Ic, PopA3], distributed mainly in Northern, Southern, and Western Africa, respectively. Tanaka *et al.* (2013) identified melon accessions with Ic cytoplasm only in the African continent, mainly in Western Africa but with a few in Southern Africa. Although seven additional accessions from Africa were examined in this study, all were of Ia or Ib type, and thus, so far, Ic type appears to be endemic to Western and Southern Africa. These results indicate distinct genetic differentiation among three groups of melon, subsp. *melo* and the Ia and Ic types of subsp. *agrestis*, indicating nearly perfect divergence in both nuclear and chloroplast genomes. The MJ network, NJ tree, and ML tree based on SNPs in the chloroplast genome place the Ic type apart from the Ia and Ib types (Fig. 3, Supplemental Fig. 1). Endl *et al.* (2018) located Tibish, a unique type of vegetable melon endemic to Sudan, in the most basal clade within *C. melo*, consisting exclusively of African accessions, and described this clade as subsp. *meloides*, which was considered the wild progenitor of Tibish and Fadasi. Among seven accessions of [small seed, Ic, PopA3] and three accessions of [small seed, Ic, admixture PopA1/A3], five were cultivated and five were wild or feral (Supplemental Table 1). A Ghanaian accession, PI 185111, classified as subsp. *meloides* by Endl *et al.* (2018), was identified as a [small seed, Ic, admixture PopA1/A3] type (Supplemental Table 1), suggesting a close relationship between subsp. *meloides* and Ic type accessions of subsp. *agrestis*. The Ic type melon was considered to be domesticated from the Ic type wild melon subsp. *meloides* in Africa. Although Ic type melons such as Tibish and Fadasi are grown only in limited areas of Africa, they have a unique nuclear genome of PopA3 and thus could be of considerable potential value as unique genetic resources for breeding. Further studies including genome wide SNP analysis and whole sequencing of nuclear and chloroplast genomes should be carried out using more accessions especially from Sub-Saharan Africa where Ic type melon distributes.

## Author Contribution Statement

KT and KK conceived the project; KK provided materials; KT, TPD, PTPN, and MT performed the experiments; KT analyzed the data, prepared figures, and wrote a draft of the manuscript; and GS, YM, HN, and RI provided advice on the experimental implementation and helped draft the manuscript.

## Supplementary Material

Supplemental Figures

Supplemental Tables

## Figures and Tables

**Table 1.** PCR primers used for genotyping the chloroplast genome and for sequencing target regions

Marker	Primers (5ʹ to 3ʹ) (F; Forward, R; Reverse)*^a^*		Gene or intergenic region*^b^*	Polymorphism			Restriction enzyme
				Type*^c^*	Sequence*^c^*	Position (bp)*^b^*	
Chloroplast genotyping							
Cmcp1-dCAPS	F	AATATCCAAATACCAAATT**g**T	*matK*	SNP2	C/A	1,974	*Rsa*I
	R	TCGGAATTATTGGAAGAATTCTT					
Cmcp2-CAPS	F	CAAAGAATCATATAAATAATGG	*rps16* intron1	SNP6	A/C	5,728	*Apo*I
	R	CGAAAGGATCCAATTCGAACAAG					
Cmcp3-dCAPS	F	TATTGAAATTATTCTA**c**AGCT	*psbK-psbI*	SNP8	G/A	8,170	*Pvu*II
	R	CTCGAAGAGGGTCGTTCAAAG					
PSID-dCAPS1	F	AAAAAAAAACAATTGCAGAT**a**T	*rpl16*–*rpl14*	SNP17	C/A	83,144	*Eco*RV
PSID-dCAPS2	F	AAAAAAAAACAATTGCAGATT**r**A**a**TT		SNP18	T/A	83,140	*Apo*I
	R	TGCAGCATTTAAAAGGGTCTGAGGT					
Cmcp6-dCAPS	F	GTAAAATTTTTTGACAAT**t**TA	*ndhF*–*rpl32*	SNP19	G/A	114,625	*Dra*I
	R	AATTATTCTTTCTTGCTCTAG					
Cmcp11-dCAPS	F	AGAATACTATAAGATAAAAAT**g**TA	*ndhA* intron1	SNP25	C/G/18 bp del.	122,326	*Rsa*I
	R	ATAACAGACAGAATTCTATTG					
Cmcp11-CAPS1	F	AAACTTAAAGCCCCAATTCGT	*ndhA* intron1	SNP29	T/G	122,870	*Hae*III
	R	TTTTCAAAATTATCCAAAACC					
Cmcp11-CAPS2	F	GGGCTCTCTTGCGCCTATATT	*ndhA* intron1	SNP30	C/T	123,140	*Hin*fI
	R	CAGCTTATATAAATAAAAATTAGC					
ccSSR-7	F	CGGGAAGGGCTCGKGCAG	*psbC*–*trnS*	InDel1	5 bp del.	37,347–37,351	–
	R	GTTCGAATCCCTCTCTCTCCTTTT					
Cmcp3-SSR	F	TGTTTGGCAAGCTGCTGTAAG	*psbK–psbI*	SSR4	(A)_12–17_	8,072–8,083	–
	R	TGGATTCAACTTAAAGCTTCAGAC					
PSID-SSR	F	CGAACCCAATTCATTACTTCGG	*rpl16*–*rpl14*	SSR6	(T)_10–12_	83,129–83,139	–
	R	AAAAGAAATATTGTTTTTCAAA					
Sequencing							
Cmcp3	F	TGTTTGGCAAGCTGCTGTAAG	*psbK–psbI*	–	–	–	–
	R	GAGAGTAAGCATTACACAATCTCCAAG					
PSID	A	AAAGATCTAGATTTCGTAAACAACATAGAGGAAGAA	*rpl16*–*rpl14*	–	–	–	–
	B	ATCTGCAGCATTTAAAAGGGTCTGAGGTTGAATCAT					
Cmcp11	F1	TATAGGTTGACGCCACAAATTC	*ndhA* intron1	–	–	–	–
	R2	GATATTTTTTATTGTTTCTTATCTCC					

*^a^* R = A or G, K = T or G. *^b^* Marker position indicated by gene name and physical position in melon chloroplast genome (accession No. JF412791). *^c^* Type of polymorphism cited from Tanaka *et al.* (2013).

**Table 2.** Chloroplast genome types discriminated by the analysis of 12 diagnostic markers

Cytoplasm type/Species*^a^*	Number of accessions	SNP2		SNP6		SNP8		SNP17		SNP18		SNP19		SNP25		SNP29		SNP30		InDel1		SSR4		SSR6
		Cmcp1-dCAPS		Cmcp2-CAPS		Cmcp3-dCAPS		PSID-dCAPS1		PSID-dCAPS2		Cmcp6-dCAPS		Cmcp11-dCAPS		Cmcp11-CAPS1		Cmcp11-CAPS2		cc SSR7		Cmcp3-SSR2		PSID-SSR
Ia-1	86	C		C		G		A		A		A		G		G		T		–		(A)17		(T)11
Ia-2	2	C		C		G		A		A		A		C		T		T		–		(A)17		(T)11
Ia-3	23	C		C		G		A		A		A		C		T		T		–		(A)16		(T)11
Ia-4	5	C		C		G		A		A		A		Deletion		T		T		–		(A)16		(T)11
Ia-5	1	C		C		G		A		A		A		Deletion		T		T		–		(A)16		(T)12
Ia-6	1	C		C		G		A		A		A		C		T		T		–		(A)15		(T)11
Ib-1	24	A		C		A		A		T		G		C		T		T		–		(A)15		(T)11
Ib-2	36	A		C		A		A		T		G		C		T		T		–		(A)15		(T)10
Ib-3	22	A		C		A		A		T		G		C		T		T		Deletion		(A)15		(T)11
Ic-1	1	C		A		G		C		T		G		Deletion		T		C		–		(A)13		(T)11
Ic-2	1	C		A		G		C		T		G		C		T		C		–		(A)15		(T)11
Ic-3/-4*^b^*	4	C		A		G		C		T		G		C		T		C		–		(A)14		(T)11
Ic-5/-6*^c^*	6	C		A		G		C		T		G		C		T		C		–		(A)12		(T)12
*C. anguria*		C		A		G		A		T		G		Deletion		T		C		–		(A)14		(T)11
*C. hystrix*		C		A		G		C		T		G		Deletion		T		C		–		(A)13		(T)11
*C. metuliferus*		C		A		G		C		T		G		Deletion		T		C		–		(A)14		(T)5
*C. sagittatus*		C		A		G		C		T		Deletion		Deletion		T		C		–		(A)11		(T)8
Total	212																							

*^a^* Sequence data was cited from Tanaka *et al.* (2013), with the exception of Ia-4, Ia-5 and Ia-6 which were sequenced in this study. *^b^* Ic-3 and Ic-4 show identical genotype, since SNP21 (Tanaka *et al.* 2013) is not analyzed in this study. *^c^* Ic-5 and Ic-6 show identical genotype, since InDel2 (Tanaka *et al.* 2013) is not analyzed in this study.

**Table 3.** Association between three different classifications based on seed length and polymorphisms in chloroplast and nuclear genomes

Area/Seed size	Number of accessions	Ia*^a^*							Ib*^a^*							Ic*^a^*						Gene diversity
		A1	A2	A3	B1	B2	AD		A1	A2	A3	B1	B2	AD		A1	A2	A3	B1	B2	AD	
Europe, USA*^b^*																						0.308
Large seed	27	–	–	–	–	–	–		16	11	–	–	–	(1)		–	–	–	–	–	–	
Small seed	1	–	–	–	–	–	–		–	1	–	–	–	–		–	–	–	–	–	–	
West and Central Asia																						0.345
Large seed	20	–	2	–	1	–	–		13	3	–	1	–	(2)		–	–	–	–	–	–	
Small seed	2	–	–	–	2	–	(1)		–	–	–	–	–	–		–	–	–	–	–	–	
East Asia																						0.171
Large seed	0	–	–	–	–	–	–		–	–	–	–	–	–		–	–	–	–	–	–	
Small seed	33	–	–	–	1	31	–		–	–	–	–	1	–		–	–	–	–	–	–	
South Asia																						0.334
Large seed	23	2	3	–	5	–	–	0	6	3	–	4	–	(2)		–	–	–	–	–	–	
Small seed	46	1	5	–	35	1	(2)	0	2	–	–	2	–	–		–	–	–	–	–	–	
Southeast Asia																						0.261
Large seed	6	–	–	–	3	–	–		2	1	–	–	–	–		–	–	–	–	–	–	
Small seed	16	2	–	–	11	2	(1)		1	–	–	–	–	–		–	–	–	–	–	–	
Africa																						0.312
Large seed	11	1	–	–	–	–	–		7	3	–	–	–	–		–	–	–	–	–	–	
Small seed	15	–	–	1	5	–	–		–	–	–	–	–	–		–	–	7	2	–	(3)	
Total	200	6	10	1	63	34	(4)		47	22	–	7	1	(5)		–	–	7	2	–	(3)	

*^a^* Ia, Ib and Ic indicate cytoplasm type. A1, A2, A3, B1 and B2 indicate model-based subpopulation, and AD indicates admixture type. No. of admixture accessions indicated in parentheses. *^b^* East Asian Cantalupensis and Inodorus are included. Five types representing Europe/US large seed-type, East Asian small seed-type, and Ic type of Africa are highlighted in green, pink, and blue, respectively.
