# Supplementary material for: Polyphyletic domestication and inter-lineage hybridization magnified genetic diversity of cultivated melon, Cucumis melo L
Source: Breed Sci. 2025 Jun 10;75(3):168–78. doi: 10.1270/jsbbs.24045 (PMC12457790; doi:10.1270/jsbbs.24045)
Supplement: Supplementary file 2 — Supplemental Tables [file 75_168_s2.pdf]

Supplemental Table 1. List of melon accessions analysed in this study

Cultivar name / Accession number	Seed source [c]	Geographical group	Horticultural group	Cultivar group / Country	Seed type [d]	Chloroplast genome type	Cluster ID (Fig. 5A)	Membership (STRUCTURE)
Melon Cantalupo di Charentais	1	Europe/US	Cantalupensis	Europian cantaloup	L	Ib-1	IIc	Pop A2
Cantaloure de Bellegrade [a]	1	Europe/US	Cantalupensis	Europian cantaloup	L	Ib-1	IIc	Pop A2
Ogen (780045) [a]	1	Europe/US	Cantalupensis	Europian cantaloup	L	Ib-1	Ic	Pop A1
Barnett Hill Favourite	1	Europe/US	Cantalupensis	England greenhouse type	L	Ib-2	IIb	Pop A1
Earl's Favorite	1	Europe/US	Cantalupensis	England greenhouse type	L	Ib-2	Ib	Pop A1
Blenheim Orange	1	Europe/US	Cantalupensis	England greenhouse type	L	Ib-2	Ib	Pop A1
British Queen	1	Europe/US	Cantalupensis	England greenhouse type	L	Ib-2	Ib	Pop A1
Hero of Lockinge	1	Europe/US	Cantalupensis	England greenhouse type	L	Ib-2	IIb	Pop A2
Homegarden	1	Europe/US	Cantalupensis	American field type	L	Ib-3	VII	Pop A2
Georgia 47	1	Europe/US	Cantalupensis	American field type	L	Ib-2	V	Pop A2/A3
Rocky Ford	1	Europe/US	Cantalupensis	American field type	L	Ib-1	IIc	Pop A2
♯58-21	1	Europe/US	Cantalupensis	American field type	L	Ib-3	IIc	Pop A2
SC108 (C-108)	1	Europe/US	Cantalupensis	American field type	L	Ib-2	Ia	Pop A1
Rio Gold	1	Europe/US	Cantalupensis	American field type	L	Ib-2	IIc	Pop A2
Hale's Best	1	Europe/US	Cantalupensis	American field type	L	Ib-2	IIc	Pop A2
Spicy	1	Europe/US	Cantalupensis	American field type	L	Ib-3	IIc	Pop A2
Kurume 2	1	Europe/US	Cantalupensis	Japan breeding line	L	Ib-2	Ib	Pop A1
Melon Chuukanbohon Nou 1	1	Europe/US	Cantalupensis	Japan breeding line	S	Ib-2	IIb	Pop A2
Honeydew	1	Europe/US	Inodorus	Honeydew	L	Ib-3	Ia	Pop A1
600011	1	Europe/US	Inodorus	Haneydew	L	Ib-3	Ia	Pop A1
610002	1	Europe/US	Inodorus	Haneydew	L	Ib-3	Ia	Pop A1
650013	1	Europe/US	Inodorus	Haneydew	L	Ib-3	Ia	Pop A1
Chinese Honeydew	1	Europe/US	Inodorus	Chinese Honeydew	L	Ib-3	Ia	Pop A1
Carosello Scopatizzo Barese	1	Europe/US	Inodorus	Spain	L	Ib-2	Ia	Pop A1
Spain Noboru 3	1	Europe/US	Inodorus	Spain	L	Ib-3	IIb	Pop A2
Tendral [b]	1	Europe/US	Inodorus	Spain	L	Ib-3	IIb	Pop A2
Kokand	1	Europe/US	Inodorus	Russia	L	Ib-3	Ia	Pop A1
Mirzuchulskaja	1	Europe/US	Inodorus	Russia	L	Ib-3	Ia	Pop A1
Ak-Urug	1	Europe/US	Inodorus	Russia	L	Ib-3	Ia	Pop A1
Hami-gua 6	1	West and Central Asia	Inodorus	Chinese Hami melon	L	Ib-2	Ia	Pop A1
Hami-gua	1	West and Central Asia	Inodorus	Chinese Hami melon	L	Ib-2	Ia	Pop A1
Hami-gua 2	1	West and Central Asia	Inodorus	Chinese Hami melon	L	Ib-2	Ia	Pop A1
Hami-gua H	1	West and Central Asia	Inodorus	Chinese Hami melon	L	Ib-2	Ia	Pop A1
Hami-gua J	1	West and Central Asia	Inodorus	Chinese Hami melon	L	Ib-2	Ia	Pop A1
940068	1	West and Central Asia	Agrestis	Iran	S	Ia-3	VII	Pop A2/A3
940069	1	West and Central Asia	Agrestis	Iran	S	Ia-3	VII	Pop B1
Karasu melon	1	West and Central Asia	Unclassified landrace	Turkey	L	Ib-2	VII	Pop A2
PI 169379	2	West and Central Asia	Unclassified landrace	Turkey	L	Ib-1	Ia	Pop A1
PI 172818	2	West and Central Asia	Unclassified landrace	Turkey	L	Ia-3	IIc	Pop A2
PI 176928	2	West and Central Asia	Unclassified landrace	Turkey	L	Ib-2	Ia	Pop A1
PI 181872	2	West and Central Asia	Unclassified landrace	Syria	L	Ib-2	Ia	Pop A1
PI 534605	2	West and Central Asia	Unclassified landrace	Syria	L	Ib-1	Ia	Pop A1
PI 181747	2	West and Central Asia	Unclassified landrace	Lebanon	L	Ib-2	Ia	Pop A1
PI 369161	2	West and Central Asia	Unclassified landrace	Lebanon	L	Ia-1	VII	Pop B1
PI 435286	2	West and Central Asia	Unclassified landrace	Iraq	S	Ia-3	V	Pop B1
PI 435290	2	West and Central Asia	Unclassified landrace	Iraq	L	Ib-2	VII	Pop A1/A2
PI 140814	2	West and Central Asia	Unclassified landrace	Iran	L	Ib-3	Ia	Pop A1
PI 143231	2	West and Central Asia	Unclassified landrace	Iran	L	Ib-3	VII	Pop A2
PI 230185	2	West and Central Asia	Unclassified landrace	Iran	L	Ib-2	VI-c	Pop B1
PI 126054	2	West and Central Asia	Unclassified landrace	Afghanistan	L	Ia-3	IIb	Pop A2
PI 125931	2	West and Central Asia	Unclassified landrace	Afghanistan	L	Ib-3	IIb	Pop A2
PI 125961	2	West and Central Asia	Unclassified landrace	Afghanistan	L	Ib-1	Ic	Pop A1
PI 126047	2	West and Central Asia	Unclassified landrace	Afghanistan	L	Ib-2	Ia	Pop A1/A3
PI 127532	2	West and Central Asia	Unclassified landrace	Afghanistan	L	Ib-1	Ic	Pop A1
PI 614375	2	South Asia	Flexuosus	India-west	L	Ia-1	Ib	Pop A1
PI 614543	2	South Asia	Flexuosus	India-center	L	Ia-1	IIc	Pop A2
PI 614576	2	South Asia	Flexuosus	India-west	L	Ia-1	IIc	Pop A2
NP20	3	South Asia	Flexuosus	India-north	S	Ia-1	IIc	Pop A2
PI 614543	2	South Asia	Flexuosus	India-center	L	Ia-1	IIc	Pop B1
NP12	3	South Asia	Flexuosus	India-east	L	Ia-1	IIc	Pop B1
PI 182952	2	South Asia	Momordica	India-west	L	Ib-1	VI-c	Pop B1
NP5	3	South Asia	Momordica	India-north	S	Ia-1	VI-c	Pop B1
PI 116479	2	South Asia	Momordica	India-north	L	Ia-1	III	Pop A1
NP14	3	South Asia	Momordica	India-north	L	Ib-2	Ib	Pop A1
PI 614556	2	South Asia	Momordica	India-center	L	Ib-1	IIa	Pop A2
PI 124096	2	South Asia	Momordica	India-south	L	Ib-1	VI-c	Pop B1
PI 210077	2	South Asia	Momordica	India-east	S	Ia-1	VI-b	Pop B1/B2
PI 124208	2	South Asia	Momordica	India-east	L	Ia-1	VII	Pop A2
940262	1	South Asia	Momordica	Nepal	S	Ia-1	VII	Pop B1
940263	1	South Asia	Momordica	Nepal	S	Ib-1	Ib	Pop A1
940275	1	South Asia	Momordica	Bangladesh	S	Ia-3	VI-c	Pop B1
790113	1	South Asia	Momordica	Bangladesh	L	Ia-1	Ib	Pop B1
760031	1	South Asia	Momordica	Bangladesh	L	Ia-1	VII	Pop B1
940099	1	South Asia	Agrestis	Pakistan	S	Ia-4	VII	Pop B1
940101	1	South Asia	Agrestis	Pakistan	S	Ia-4	VI-c	Pop B1

940102	1	South Asia	Agrestis	Pakistan	S	Ia-5	VII	Pop A1/A2
940103	1	South Asia	Agrestis	Pakistan	S	Ia-4	VII	Pop B1
PI 164796	2	South Asia	Agrestis	India-west	S	Ia-3	III	Pop B1
PI 614433	2	South Asia	Agrestis	India-west	S	Ia-3	VI-c	Pop B1
NP10	3	South Asia	Agrestis	India-north	S	Ia-1	VI-c	Pop B1
PI 614519	2	South Asia	Agrestis	India-center	S	Ia-6	VI-c	Pop B1
PI 614549	2	South Asia	Agrestis	India-center	S	Ia-1	IIc	Pop B1
PI 536481	2	South Asia	Agrestis	Maldives	S	Ia-3	VI-c	Pop B1
820003	1	South Asia	Agrestis	Nepal	S	Ia-3	VII	Pop B1
940097	1	South Asia	Agrestis	Nepal	S	Ia-3	VI-c	Pop B1
940109	1	South Asia	Agrestis	Nepal	S	Ia-3	VI-c	Pop B1
770134	1	South Asia	Agrestis	Bangladesh	S	Ia-4	VI-c	Pop B1
770135	1	South Asia	Agrestis	Bangladesh	S	Ia-4	VI-c	Pop B1
790112	1	South Asia	Agrestis	Bangladesh	S	Ia-3	VI-c	Pop B1
PI 116738	2	South Asia	Unclassified landrace	India-west	S	Ib-2	VI-c	Pop B1
PI 614262	2	South Asia	Unclassified landrace	India-west	S	Ia-3	VI-c	Pop B1
PI 116666	2	South Asia	Unclassified landrace	India-west	L	Ia-1	IIa	Pop B1
PI 164825	2	South Asia	Unclassified landrace	India-west	L	Ib-1	IV	Pop A1/A3
PI 175109	2	South Asia	Unclassified landrace	India-north	S	Ia-1	V	Pop B1
PI 179666	2	South Asia	Unclassified landrace	India-north	S	Ib-1	III	Pop A1
NP2	3	South Asia	Unclassified landrace	India-north	L	Ib-1	Ib	Pop A1
PI 124109	2	South Asia	Unclassified landrace	India-north	L	Ib-1	Ia	Pop A1
PI 165508	2	South Asia	Unclassified landrace	India-north	S	Ia-1	IIa	Pop A2
PI 614542	2	South Asia	Unclassified landrace	India-center	S	Ia-1	VI-a	Pop B1
PI 614561	2	South Asia	Unclassified landrace	India-center	S	Ia-2	VI-c	Pop B1
PI 614566	2	South Asia	Unclassified landrace	India-center	S	Ia-1	VII	Pop A2
PI 614567	2	South Asia	Unclassified landrace	India-center	S	Ia-1	VI-c	Pop B1
PI 614568	2	South Asia	Unclassified landrace	India-center	S	Ia-1	VI-a	Pop B1
PI 124435	2	South Asia	Unclassified landrace	India-center	L	Ib-2	V	Pop B1
PI 614588	2	South Asia	Unclassified landrace	India-center	S	Ia-2	VI-c	Pop B1
PI 164323	2	South Asia	Unclassified landrace	India-south	S	Ia-1	VI-a	Pop B1
PI 164585	2	South Asia	Unclassified landrace	India-south	L	Ib-2	III	Pop A1
PI 123684	2	South Asia	Unclassified landrace	India-south	L	Ib-3	Ib	Pop A1
PI 124105	2	South Asia	Unclassified landrace	India-south	L	Ib-3	Ic	Pop A1
PI 123501	2	South Asia	Unclassified landrace	India-south	L	Ib-2	IIc	Pop A2
PI 124113	2	South Asia	Unclassified landrace	India-east	S	Ia-1	III	Pop A1
PI 124112	2	South Asia	Unclassified landrace	India-east	S	Ib-1	VII	Pop B1
PI 166125	2	South Asia	Unclassified landrace	India-east	S	Ia-1	VI-a	Pop B1
PI 124111	2	South Asia	Unclassified landrace	India-east	L	Ib-1	IIa	Pop A2
PI 124207	2	South Asia	Unclassified landrace	India-east	L	Ib-2	III	Pop A1/A2
PI 124112	2	South Asia	Unclassified landrace	India-east	L	Ib-1	VII	Pop B1
PI 210541	2	South Asia	Unclassified landrace	India-east	S	Ia-1	VII	Pop A2
PI 210542	2	South Asia	Unclassified landrace	India-east	S	Ia-1	VI-b	Pop B2
PI 210541	2	South Asia	Unclassified landrace	India-east	S	Ia-1	VII	Pop A2
PI 536479	2	South Asia	Unclassified landrace	Maldives	S	Ia-1	VI-c	Pop B1
PI 536480	2	South Asia	Unclassified landrace	Maldives	S	Ia-1	VII	Pop B1
NP1	3	South Asia	Unclassified landrace	Nepal	S	Ia-1	VII	Pop B1
790079	1	South Asia	Unclassified landrace	Bangladesh	S	Ia-1	VII	Pop B1
Joydebpul	1	South Asia	Unclassified landrace	Bangladesh	S	Ia-1	VII	Pop B1
C5-3	1	South Asia	Unclassified landrace	Bangladesh	S	Ia-1	VI-c	Pop B1
890096	1	South Asia	Unclassified landrace	Bangladesh	S	Ia-1	Ib	Pop B1
940269	1	South Asia	Unclassified landrace	Bangladesh	S	Ia-3	VI-c	Pop B1
910050	1	Southeast Asia	Flexuosus	Indonesia	L	Ib-1	Ib	Pop A1
940261	1	Southeast Asia	Momordica	Myanmar	S	Ia-1	Ib	Pop A1
650057	1	Southeast Asia	Momordica	Myanmar	L	Ia-1	VI-c	Pop B1
My238	3	Southeast Asia	Unclassified landrace	Myanmar	S	Ia-1	VI-b	Pop B2
PI 200814	2	Southeast Asia	Unclassified landrace	Myanmar	S	Ia-1	VI-c	Pop B1
PI 200816	2	Southeast Asia	Unclassified landrace	Myanmar	S	Ia-1	VI-c	Pop B1
PI 200817	2	Southeast Asia	Unclassified landrace	Myanmar	S	Ia-1	VI-c	Pop B1
PI 200819	2	Southeast Asia	Unclassified landrace	Myanmar	S	Ia-1	VI-c	Pop B1
PI 200813	2	Southeast Asia	Unclassified landrace	Myanmar	L	Ia-1	V	Pop B1
940300	1	Southeast Asia	Unclassified landrace	Thailand	S	Ia-3	VI-c	Pop B1
940301	1	Southeast Asia	Unclassified landrace	Thailand	S	Ia-3	VI-c	Pop B1
940303	1	Southeast Asia	Unclassified landrace	Thailand	S	Ia-1	VI-c	Pop B1
940320	1	Southeast Asia	Unclassified landrace	Thailand	S	Ib-1	Ib	Pop A1
T3	3	Southeast Asia	Unclassified landrace	Thailand	S	Ia-1	VI-c	Pop B1
860314	1	Southeast Asia	Unclassified landrace	Thailand	S	Ia-1	Ib	Pop A1
940289	1	Southeast Asia	Unclassified landrace	Malaysia	S	Ia-1	VI-c	Pop B1
Horimatsu	1	Southeast Asia	Unclassified landrace	Malaysia	L	Ib-2	Ia	Pop A1
B1	3	Southeast Asia	Unclassified landrace	Indonesia	S	Ia-1	VI-c	Pop B1
B2	3	Southeast Asia	Unclassified landrace	Indonesia	S	Ia-1	VI-b	Pop B1/B2
BL1	3	Southeast Asia	Unclassified landrace	Indonesia	L	Ib-3	IIb	Pop A2
MO-1	1	Southeast Asia	Unclassified landrace	Laos	S	Ia-1	VI-c	Pop B1
DGT	3	Southeast Asia	Unclassified landrace	Viet Nam	L	Ia-1	VI-c	Pop B1
DHK	3	Southeast Asia	Unclassified landrace	Viet Nam	S	Ia-1	VI-b	Pop B2
C32	3	East Asia	Conomon	China	S	Ia-1	VI-b	Pop B2
940182	1	East Asia	Conomon	China	S	Ia-3	VI-b	Pop B2
P169-1	3	East Asia	Conomon	China	S	Ia-1	VI-b	Pop B2
P171-1	3	East Asia	Conomon	China	S	Ia-1	VI-b	Pop B2
C28	3	East Asia	Makuwa	China	S	Ia-1	VI-b	Pop B2

Mi-tang-tin	1	East Asia	Makuwa	China	S	Ia-1	VI-b	Pop B2
760007	1	East Asia	Makuwa	China	S	Ia-3	VI-b	Pop B2
910008	1	East Asia	Makuwa	China	S	Ia-1	VI-b	Pop B2
780143	1	East Asia	Makuwa	China	S	Ia-1	VI-b	Pop B2
910055	1	East Asia	Makuwa	China	S	Ia-1	VI-b	Pop B2
940178	1	East Asia	Makuwa	China	S	Ia-1	VI-b	Pop B2
940184	1	East Asia	Makuwa	China	S	Ia-1	VI-b	Pop B2
Chi-87-12	1	East Asia	Makuwa	China	S	Ia-1	VI-b	Pop B2
PI 136173	2	East Asia	Makuwa	China	S	Ia-1	VI-c	Pop B1
PI 157070	2	East Asia	Makuwa	China	S	Ia-1	VI-b	Pop B2
630044	1	East Asia	Makuwa	Korea	S	Ia-1	VI-b	Pop B2
630047	1	East Asia	Makuwa	Korea	S	Ia-1	VI-b	Pop B2
940147	1	East Asia	Makuwa	Korea	S	Ib-1	VI-b	Pop B2
Takada-shiro-uri	1	East Asia	Conomon	Japan	S	Ia-1	VI-b	Pop B2
Tokyo-wase-shiro-uri	1	East Asia	Conomon	Japan	S	Ia-1	VI-b	Pop B2
Nakasaki-tsuke-uri	1	East Asia	Conomon	Japan	S	Ia-1	VI-b	Pop B2
Karimori	1	East Asia	Conomon	Japan	S	Ia-1	VI-b	Pop B2
Hyougo-aoshima-uri	1	East Asia	Conomon	Japan	S	Ia-1	VI-b	Pop B2
Kanro	1	East Asia	Makuwa	Japan	S	Ia-1	VI-b	Pop B2
Kinpyo	1	East Asia	Makuwa	Japan	S	Ia-1	VI-b	Pop B2
Seikan	1	East Asia	Makuwa	Japan	S	Ia-1	VI-b	Pop B2
Nanbukin	1	East Asia	Makuwa	Japan	S	Ia-1	VI-b	Pop B2
Kohimeuri	3	East Asia	Makuwa	Japan	S	Ia-1	VI-b	Pop B2
Weedy melon	3	East Asia	Agrestis	Korea	S	Ia-3	VI-b	Pop B2
940082	1	East Asia	Agrestis	Korea	S	Ia-3	VI-b	Pop B2
940009	1	East Asia	Agrestis	Japan	S	Ia-1	VI-b	Pop B2
940012	1	East Asia	Agrestis	Japan	S	Ia-1	VI-b	Pop B2
940047	1	East Asia	Agrestis	Japan	S	Ia-1	VI-b	Pop B2
940111	1	Western Africa	Agrestis	Sudan	S	Ic-5/-6	IV	Pop A3
PI 185111	2	Western Africa	Agrestis	Ghana	S	Ic-1	IV	Pop A1/A3
940108	1	Western Africa	Agrestis	Senegal	S	Ic-3/-4	IV	Pop B1
940112	1	Western Africa	Agrestis	Senegal	S	Ic-3/-4	IV	Pop B1
PI 436532	2	Western Africa	Agrestis	Senegal	S	Ic-3/-4	IV	Pop A1/A3
PI 436534	2	Western Africa	Agrestis	Senegal	S	Ic-3/-4	IV	Pop A3
940065	1	Western Africa	Agrestis	Cameroon	S	Ic-5/-6	IV	Pop A3
940107	1	Southern Africa	Agrestis	South Africa	S	Ia-1	VI-c	Pop B1
PI 525105	2	Northern Africa	Unclassified landrace	Egypt	L	Ib-1	Ia	Pop A1
PI 525109	2	Northern Africa	Unclassified landrace	Egypt	L	Ib-2	Ia	Pop A1
PI 525110	2	Northern Africa	Unclassified landrace	Egypt	L	Ib-2	Ia	Pop A1
PI 525111	2	Northern Africa	Unclassified landrace	Egypt	L	Ib-2	Ia	Pop A1
PI 525114	2	Northern Africa	Unclassified landrace	Egypt	L	Ib-2	Ia	Pop A1
PI 527386	2	Northern Africa	Unclassified landrace	Algeria	L	Ib-3	Ia	Pop A1
PI 207658	2	Northern Africa	Unclassified landrace	Morocco	L	Ib-1	IIc	Pop A2
PI 207661	2	Northern Africa	Unclassified landrace	Morocco	L	Ib-3	IIa	Pop A2
PI 490388	2	Western Africa	Unclassified landrace	Mali	L	Ib-2	VII	Pop A2
940281	1	Western Africa	Unclassified landrace	Chad	S	Ic-5/-6	IV	Pop A3
PI 436533	2	Western Africa	Unclassified landrace	Senegal	S	Ic-2	Ic	Pop A1/A3
PI 320993	2	Western Africa	Unclassified landrace	Sierra Leone	L	Ia-1	Ib	Pop A1
Cam-84-3	1	Western Africa	Unclassified landrace	Cameroon	S	Ic-5/-6	IV	Pop A3
PI 505599	2	Southern Africa	Unclassified landrace	Zambia	S	Ia-1	IV	Pop A3
PI 505602	2	Southern Africa	Unclassified landrace	Zambia	S	Ia-1	VI-c	Pop B1
PI 482398	2	Southern Africa	Unclassified landrace	Zimbabwe	S	Ic-5/-6	IV	Pop A3
PI 482411	2	Southern Africa	Unclassified landrace	Zimbabwe	S	Ia-1	VII	Pop B1
PI 482413	2	Southern Africa	Unclassified landrace	Zimbabwe	S	Ic-5/-6	IV	Pop A3
PI 482424	2	Southern Africa	Unclassified landrace	Zimbabwe	S	Ia-3	VII	Pop B1
PI 482429	2	Southern Africa	Unclassified landrace	Zimbabwe	S	Ia-3	VII	Pop B1
PI 234607	2	Southern Africa	Unclassified landrace	South Africa	L	Ib-3	Ic	Pop A1

[a] Two cultivars listed as ‘Ogen’ and ‘Cantaloure de Bellegrade’ are considered as ‘Ha'Ogen’ and ‘Cantaloup de Bellegarde’, respectively.
[b] Tendral o Invernale a Buccia Verde.
[c] Seed source is indicated by numbers: 1 = NARO Institute of Vegetable and Floriculture Science (NARO/NIVFS), Japan. 2 = North Central Regional Plant Introduction Station, Iowa State University (USDA-ARS), USA. 3 = Okayama University, Japan.
[d] S: Small seed-type, L: Large seed-type.

Supplemental Table 2. Accession number of nucleotide sequence registered in the DNA Data Bank of Japan

Sequence accession number	Accession number	Cytoplasm type	Sequence region
LC822783	940102	Ia-5	Protein-coding region of *psbK* gene and *psbK–psbI* intergenic region
LC822784	PI 614519	Ia-6	Protein-coding region of *psbK* gene and *psbK–psbI* intergenic region
LC822785	940102	Ia-5	Protein-coding region of *rpl16* gene and *rpl16–rpl14* inter-genic region (PS-ID)
LC822786	PI 614519	Ia-6	Protein-coding region of *rpl16* gene and *rpl16–rpl14* inter-genic region (PS-ID)
LC822787	940099	Ia-4	Protein-coding region of *ndhA* gene and *ndhA* intron1
LC822788	940102	Ia-5	Protein-coding region of *ndhA* gene and *ndhA* intron1

Supplemental Table 3. Statistics of the 27 RAPD markers generated from 212 melon accessions

Marker name	Primer sequence (5' to 3')	Number of different alleles	Number of effective alleles	PIC
A07-1353	GATGGATTTGGG	2.0	1.9	0.126
A07-872	GATGGATTTGGG	2.0	1.5	0.171
A20-1100	TTGCCGGGACCA	2.0	2.0	0.196
A20-800	TTGCCGGGACCA	2.0	1.3	0.044
A22-1520	TCCAAGCTACCA	2.0	1.2	0.085
A23-1200	AAGTGGTGGTAT	2.0	1.5	0.235
A26-1400	GGTGAGGATTCA	2.0	1.5	0.126
A31-800	GGTGGTGGTATC	2.0	2.0	0.229
A39-2027	CCTGAGGTAACT	2.0	1.6	0.187
A41-1353	TGGTAGGTAACT	2.0	1.5	0.196
A41-1020	TGGTAGGTAACT	2.0	1.3	0.175
A41-930	TGGTAGGTAACT	2.0	2.0	0.257
A57-800	ATCATTGGCGAA	2.0	2.0	0.221
B15-600	CCTTGGCATCGG	2.0	1.9	0.209
B32-900	ATCATCGTACGT	2.0	2.0	0.252
B32-700	ATCATCGTACGT	2.0	1.8	0.223
B68-1078	CACACTCGTCAT	2.0	2.0	0.225
B71-1220	GGACCTCCATCG	2.0	1.5	0.244
B84-700	CTTATGGATCCG	2.0	1.4	0.180
B84-600	CTTATGGATCCG	2.0	1.9	0.282
B84-550	CTTATGGATCCG	2.0	1.3	0.123
B86-1500	ATCGAGCGAACG	2.0	1.2	0.076
B86-1350	ATCGAGCGAACG	2.0	1.6	0.114
B96-850	CTGAAGACTATG	2.0	1.5	0.240
B96-750	CTGAAGACTATG	2.0	1.9	0.230
B99-1400	TTCTGCTCGAAA	2.0	1.6	0.156
C00-1350	GAGTTGTATGCG	2.0	1.7	0.163

Supplemental Table 4. Genetic variation in 10 groups of melon classified by geographical origin and seed size type.

Area / Seed size	Number of accessions	Number of alleles	Number of effective alleles	Gene diversity
Europe/US_large seed	28	1.78	1.54	0.261
West and Central Asia_large seed	22	1.89	1.58	0.318
West and Central Asia_small seed	3	1.19	1.32	0.230
South Asia_large seed	25	1.93	1.67	0.367
South Asia_small seed	48	2.00	1.52	0.292
Southeast Asia_large seed	6	1.67	1.50	0.307
Southeast Asia_small seed	17	1.78	1.35	0.211
East Asia_small seed	33	1.48	1.27	0.147
Africa_large seed	11	1.59	1.52	0.223
Africa_small seed	18	1.78	1.42	0.262

Supplemental Table 5. Pairwise estimates of F_{ST} and genetic distance among five model-based subpopulations

Subpopulation	PopA1	PopA2	PopA3	PopB1	PopB2
PopA1	-	0.2144	0.5754	0.3974	0.6119
PopA2	0.1343	-	0.5340	0.2404	0.5164
PopA3	0.3528	0.3733	-	0.5948	0.6628
PopB1	0.3054	0.1517	0.3227	-	0.3462
PopB2	0.6156	0.3842	0.4550	0.1353	-

F_{ST} is shown above the diagonal, and pairwise genetic distance below the diagonal.
